# Vital signs and impaired cognition in older emergency department patients: The APOP study

**DOI:** 10.1371/journal.pone.0218596

**Published:** 2019-06-20

**Authors:** Jacinta A. Lucke, Jelle de Gelder, Laura C. Blomaard, Christian Heringhaus, Jelmer Alsma, Stephanie C. E. Klein Nagelvoort Schuit, Anniek Brink, Sander Anten, Gerard J. Blauw, Bas de Groot, Simon P. Mooijaart

**Affiliations:** 1 Department of Gerontology and Geriatrics, Leiden University Medical Center, Leiden, The Netherlands; 2 Department of Emergency Medicine, Spaarne Gasthuis, Haarlem, the Netherlands; 3 Department of Emergency Medicine, Leiden University Medical Center, Leiden, The Netherlands; 4 Department of Internal Medicine, Erasmus University Medical Center, Rotterdam, the Netherlands; 5 Department of Internal Medicine, Alrijne Hospital, Leiderdorp, The Netherlands; 6 Institute for Evidence-based Medicine in Old Age (IEMO), Leiden, The Netherlands; University of Florence, ITALY

## Abstract

**Background/Objectives:**

Cognitive impairment is a frequent problem among older patients attending the Emergency Department (ED) and can be the result of pre-existing cognitive impairment, delirium, or neurologic disorders. Another cause can also be acute disturbance of brain perfusion and oxygenation, which may be reversed by optimal resuscitation. This study aimed to assess the relationship between vital signs, as a measure of acute hemodynamic changes, and cognitive impairment in older ED patients.

**Design:**

Prospective cohort study

**Setting:**

ED’s of two tertiary care and two secondary care hospitals in the Netherlands.

**Participants:**

2629 patients aged 70-years and older

**Measurements:**

Vital signs were measured at the moment of ED arrival as part of routine clinical care. Cognition was measured using the Six-Item Cognitive Impairment Test (6-CIT).

**Results:**

The median age of patients was 78 years (IQR 74–84). Cognitive impairment was present in 738 patients (28.1%). When comparing lowest with highest quartiles, a systolic blood pressure of <129 mmHg (OR 1.30, 95% confidence interval (95%CI) 0.98–1.73)was associated with increased risk of cognitive impairment. A higher respiratory rate (>21/min) was associated with increased risk of impaired cognition (OR 2.16, 95% CI 1.58–2.95) as well as oxygen saturation of <95% (OR 1.64, 95%CI 1.24–2.19).

**Conclusion:**

Abnormal vital signs associated with decreased brain perfusion and oxygenation are also associated with cognitive impairment in older ED patients. This may partially be explained by the association between disease severity and delirium, but also by acute disturbance of brain perfusion and oxygenation. Future studies should establish whether normalization of vital signs will also acutely improve cognition.

## Introduction

Impaired cognition is a frequent problem among older patients in the Emergency Department (ED)[1, 2]. The prevalence of cognitive impairment in older ED patients is approximately 30%[3–7]. Cognitive impairment is independently associated with adverse outcome[3]. ED delirium prevalence rates of approximately 10% have been reported [8–12] and dementia was found in 3–15% of older ED patients[13–15]. Cognitive impairment in the ED can reflect pre-existing cognitive disturbance or disease (amongst which dementia), delirium and neurological disorders like encephalopathy. Alternatively, acute disturbance of brain perfusion and oxygenation due to acute hemodynamic changes or a combination of these factors may also cause acute cognitive impairment, which may quickly resolve after resuscitation. Technically this may be considered a delirium as it is an acute change in mental function as a result of a somatic disease, but the pathophysiology and disease course is different than in a classic delirium with multifactorial causes in an older patient with pneumonia. Some older ED patients may suffer from possibly reversible cognitive impairment, of which delirium is subtype, due to compromised circulation to the brain. If there is a connection between impaired brain perfusion and oxygenation due to acute hemodynamic changes and cognitive impairment in the older patients this may be a first step into investigating reversibility by optimal resuscitation in more depth.

The relationship between hemodynamic status and cognitive impairment has been investigated before[16]. There appears to be a close link between cardiac function on the one hand and cognitive functioning on the other. Regional cerebral hypoperfusion might be associated with increased risk of delirium in ICU patients[17]. Changes in cerebral blood flow cause chronic alterations to the brain, but at least some of these alterations are reversible when blood flow is restored. In patients with chronic heart failure, cognitive function improved when cardiac function improved and in patients with carotid occlusion there was a causal relationship between reduced cerebral blood flow and impaired cognition[16]. In the latter case it was proposed that cognitive impairment was caused by potentially reversible lactate accumulation in the brain[18]. But also decreased pulsatility of arterial blood flow, limited autoregulation of cerebral blood flow and chemoregulation by PaCO2 and pH could have influence[16, 19]. To our knowledge, it has never been established whether there is an association between acute short term hemodynamic changes and cognition in acutely ill older patients in the ED.

We therefore performed a multi-center prospective cohort study in which we aimed to investigate the relationship between vital signs, as a measure for acute hemodynamic changes, and the prevalence of cognitive impairment in over 2500 older ED patients.

## Methods

### Study design and setting

This was a prospective multi-center cohort study which was performed in the ED of two tertiary care and two secondary care hospitals in the Netherlands. Older patients visiting the ED of these participating hospitals were included in this study. A detailed description has been published elsewhere[20]. In short, patients were included from September 2014 –November 2014 in the Leiden University Medical Center (LUMC, Leiden), from March 2015 –June 2015 in Alrijne hospital (Alrijne, Leiderdorp), from May 2016 –July 2016 in Haaglanden Medical Center, location Bronovo (HMC Bronovo, The Hague) and from July 2016 –January 2017 in Erasmus University Medical Center (Erasmus MC, Rotterdam). Patients were included 24/7 in the LUMC hospital, 7 days a week (from 10 AM-10pm) in Alrijne Hospital, 6 days a week (from 10AM -10PM in the HMC Bronovo and 4 days a week (from 10AM-10PM) in Erasmus MC.

### Selection of participants

All patients aged 70-years and older were included consecutively. Patients who were triaged for a need of immediate care (Manchester Triage[21] category Red), patients with an unstable medical condition and patients where the doctor or nurse would felt that participating in the study would be too emotionally challenging for the patient or their relatives and therefore denied permission to enter the room, were excluded. Patients with a language barrier and patients who had a disturbed mental status according to the researcher and without a caregiver to provide informed consent were not eligible.

Patients could only be included in the study once, even if they had multiple ED visits during the study period. Two patients groups bypassed the ED and were therefore impossible to include; patients with a ST-elevation myocardial infarction were directly sent to the catheterization room; and patients with stroke and eligible for thrombolytic therapy were directly sent to the neurology ward. Written informed consent was obtained from all participants or their proxies (in case of disturbed mental status) before inclusion. The medical ethics committees of the LUMC, Alrijne Hospital, HMC Bronovo and Erasmus MC approved the study. We adhered to the STROBE guidelines.

### Methods and measurements

Teams of trained medical students included patients within 1 hour after arrival to the ED. The data collectors conducted a short 5–10 minute questionnaire on a tablet computer after which data was immediately sent to a secured database. Additional information was gathered from patient files in a standardized manner and assessed for quality by JdG. The medical students were trained by JdG to administer the questionnaires and collect data in a standardized manner.

At baseline, data on three domains were assessed: demographics, disease severity, and geriatric measurements. Demographics consisted of age, gender, living arrangement and level of education. Severity of disease consisted of characteristics related to the ED visit: way of arrival, triage category by Manchester Triage System (MTS), main complaint, fall related ED visit and vital signs. Geriatric measurements consisted of: the number of different medications stated by the patient, history of diagnosed dementia reported by patient or proxy, current use of a walking device, hours of home-care provided by a professional organisation and the Katz index of Activities of Daily Living (ADL) questionnaire. MTS category was divided into three groups, very urgent (needing treatment within 10 minutes), semi-urgent (needing treatment within 1 hour) and non-urgent (treatment can be delayed until after 1 hour).

Cognition was measured using the 6-Item Cognitive impairment Test (6-CIT) at the moment of enrolment. This short 2–3 minute test contains items on orientation, memory and concentration and has been validated[22] and used before in ED settings[15]. Scoring ranges from 0–28, with higher scores indicating more cognitive impairment. Patients with a 6-CIT score of 10 points or lower were considered to have normal cognition, those with 6-CIT ≥11 were categorized as ‘cognitive impairment’. Also patients with pre-existing dementia and those who were unable to perform the cognition test were classified as ‘impaired cognition’.

For the vital signs measurements the first set of vital signs measurements was taken from the electronic medical records, as collected by the monitor equipment of the respective hospitals. Ninety-two percent of all vital signs were measured within the first 15 minutes of ED arrival, while ninety-eight percent were measured within the first 30 minutes. Automated measured vital signs were: systolic and diastolic blood pressure (in millimetres of mercury, mmHg), heart rate (per minute), oxygen saturation (in percentage). Respiratory rate was measured automatically in LUMC and Alrijne Hospital. Respiratory rate and capillary refill time were additionally measured by hand by the data collectors in the HMC Bronovo and Erasmus MC. We chose to measure respiratory rate by hand to improve the reliability of the this measurement, as it is known to have limited accuracy when measured using standard care[23]. No other additional vital signs were measured in addition to standard care. Temperature was measured using a tympanic thermometer and manually registered in the electronic medical record by the nurse.

Laboratory test results were extracted from the electronic medical records. The first measurement during the ED visit was registered. Biochemical measures that may reflect perfusion or are essential for oxygen delivery were assessed: creatinine was measured in μmol/liter, while urea and haemoglobin were measured in mmol/liter.

### Outcome measure

The main outcome of this study was cognitive impairment, defined as a 6-CIT of 11 points or higher or the inability to perform the cognition test.

### Analysis

Patient characteristics are presented as mean with standard deviation (SD) in case of normal distribution, median with interquartile range (IQR) in case of skewed distribution or as numbers with percentages (%). Vital signs and laboratory test results (creatinine, urea, haemoglobin) were divided into quartiles. Using logistic regression the odds ratio (OR) and 95% confidence interval (95%CI) for cognitive impairment was calculated per quartile. To assess whether there was an association between vital sign quartile and cognitive impairment, the p-value for trend between quartiles was calculated using logistic regression. For the main analysis patients with pre-existing dementia (n = 142) were excluded as we wanted to assess the effect of vital signs on acute cognitive changes. Furthermore, cognition was divided into six categories: normal cognition, mild cognitive impairment (6-CIT 8–10), cognitive impairment (6-CIT 11–13), severe cognitive impairment (6-CIT ≥14), missing 6-CIT and pre-existing dementia. A 6-CIT score of ≥14 has been validated for diagnosing delirium[15], so this group of patients with severe cognitive impairment, might in fact largely represent delirium. Mean vital signs were calculated for these different categories and p-value for trend was assessed among the first four categories using linear regression. The level of significance was set at p<0.05. Statistical analyses were performed using IBM SPSS Statistics package (version 23).

### Sensitivity analyses

Two sensitivity analyses were performed. First, a similar analysis was performed excluding patients with the inability to perform the cognition test. Also patients with minor trauma (such as isolated extremity injuries, wounds and minor falls) were excluded for this analysis, since no severe acute hemodynamic changes were expected in this patient group. In a second sensitivity analysis using logistic regression we corrected for age.

In the supporting information S1 Table data on association between pulse pressure and impaired cognition is additionally shown.

## Results

**Fig 1:**
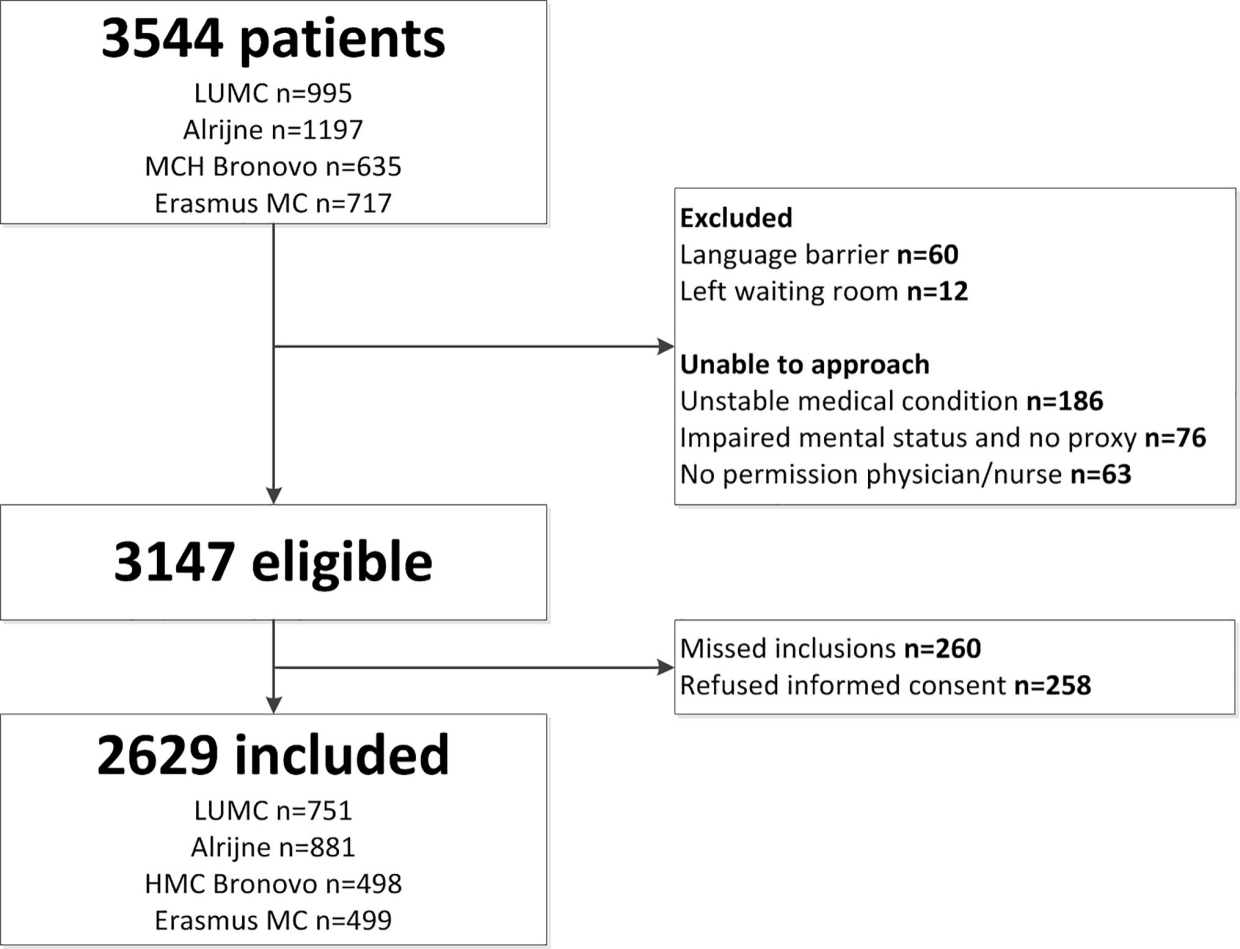
Flowchart of study population. Abbreviations: n = number.

A total of 3544 patients visited the ED of the participating hospitals during the study period, of which 3147 patients were eligible for inclusion (Fig 1), 2629 patients were included which was 83.5% of all eligible patients.

### Baseline characteristics

Table 1 shows the baseline characteristics of the study population. Median age of participants was 79 years (interquartile range (IQR) 74–84) and approximately half of them was female (n = 1393, 53.0%). A minority of patients received high education (n = 5869, 22.4%) and only a small percentage lived in a nursing home (n = 216, 8.2%). The majority of patients arrived by ambulance (n = 1339, 50.9%) and most had a problem needing medical attention within 1 hour (n = 1534, 58.3%). Mean vital signs of the study population were a systolic blood pressure of 149 mmHg(SD ±28), mean heart rate of 84/min (SD ±22) and respiratory rate of 19/min (SD ±6). The participants in this study were living relatively independent, with a median of 0 hours of home care per week (IQR 0–3 hours) and a median Katz-ADL of 0 (IQR 0–1). Cognitive impairment was found in 738 patients (28.1%).

**Table 1:** Patients characteristics of study population.

Demographics	n = 2629
Age (years), median (IQR)	79 (74–84)
Female, n (%)	1393 (53.0)
High education, n (%)	586 (22.4)
Living in a residential care/nursing home, n (%)	216 (8.2)
Hospital, n (%)	
LUMC	751 (28.6)
Alrijne	881 (33.5)
HMC Bronovo	498 (18.9)
Erasmus MC	499 (19.0)
**ED presentation characteristics**	
Arrival by ambulance, n(%)	1339 (50.9)
Triage urgency, n (%)	
> 1 hour	717 (27.3)
< 1 hour	1534 (58.3)
< 10 minutes	378 (14.4)
Fall related ED visit, n (%)	659 (25.1)
Main complaint, n(%)	
Minor	815 (31.0)
Malaise	465 (17.7)
Chest pain	393 (14.9)
Dyspnea	320 (12.2)
Abdominal pain	282 (10.7)
Other	208 (7.9)
Syncope	146 (5.6)
**Vital signs**	
Systolic BP, mmHg	149 (28)
Diastolic BP, mmHg	79 (17)
Mean Arterial Pressure, mmHg	102 (18)
Heart rate/min	84 (22)
Respiratory rate/min	19 (6)
Oxygen saturation, median (IQR)	97 (95–98)
Temperature, ⁰C	36.9 (0.9)
Capillary refill, sec, median (IQR)	2 (2–3)
**Geriatric characteristics**	
Hours of home-care, median (IQR)	0 (0–3)
Use of walking device, n (%)	1114 (42.5)
Number of medications, median (IQR)	5 (3–8)
Katz index of ADL, median (IQR)	0 (0–1)
Cognitive impairment, n (%)	738 (28.1)

Data is presented as mean, SD unless noted otherwise.

Abbreviations: n = number, % = percentage, IQR = interquartile range, ED = Emergency Department, 6CIT = 6 Item Cognitive-Impairment-Test, ADL = activities of daily living, BP = blood pressure, mmHg = millimetres of mercury, min = minute, ⁰C = degrees Celsius, sec = seconds.

Numbers between brackets indicate missing values: hours of home care (n = 72), katz baseline (n = 40), level of education (n = 16), living in nursing home (n = 1), use of walking device (n = 10), systolic BP (n = 375), diastolic BP (n = 379), mean arterial pressure (n = 379), heart rate (n = 405), respiratory rate (n = 861), oxygen saturation (n = 438), temperature (n = 716), capillary refill (n = 1705).

### Association of vital signs with impaired cognition

**Fig 2:**
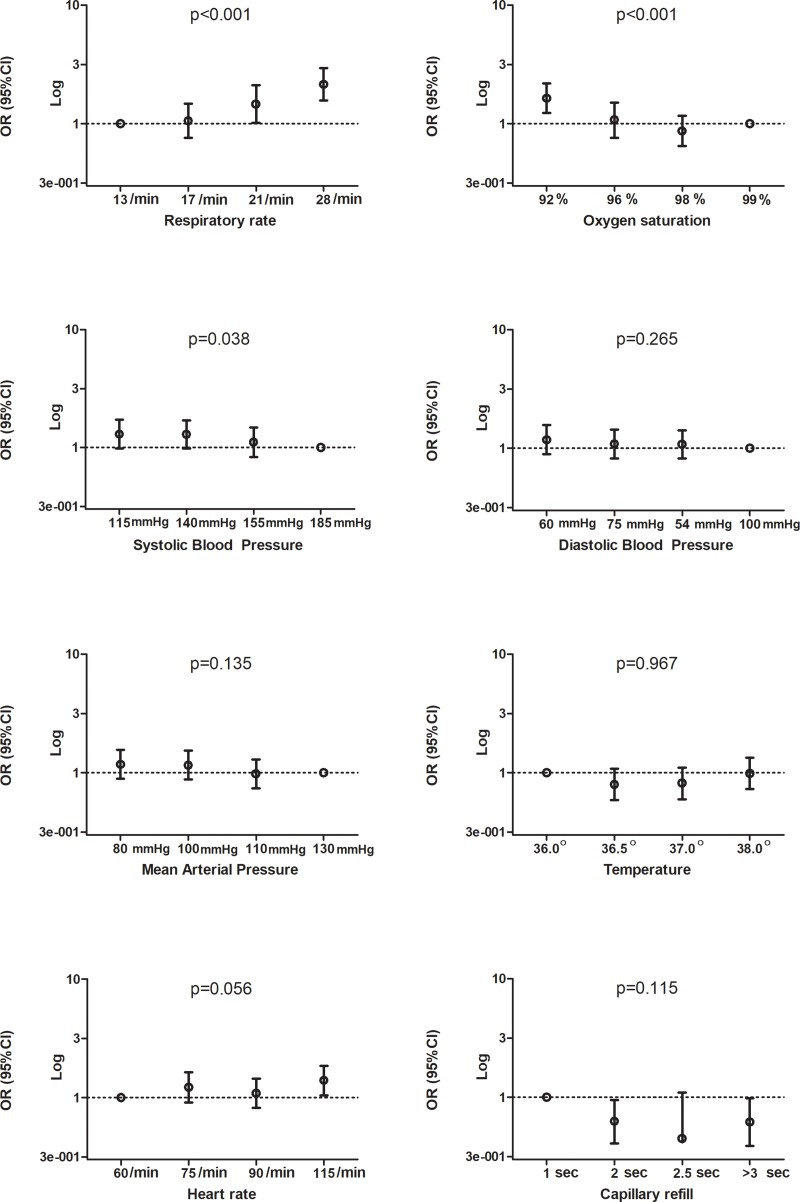
Quartiles of vital signs and their association with cognitive impairment. Abbreviations:, mmHg = millimetres of mercury, min = minute, ⁰ = degrees celsius, sec: seconds, OR = odds ratio, 95%CI = 95% confidence interval, p = p-value, % = percentage. Y-axes is a logarithmic scale. The x-axes shows quartiles of the represented vital sign with the mean value within that category as label. The circle on the dotted line represents the reference category. Odds ratios and 95% CI’s can be found in S1 Table. Dot with bars represents the odds ratio with 95% confidence interval.

Lower systolic blood pressure was associated with increased risk of impaired cognition with an OR of 1.30 (95%CI 0.98–1.73) when comparing the lowest with the highest quartile of this vital sign, as can be seen in Fig 2 and in the supporting information S1 Table. A higher respiratory rate (OR 2.16, 95%CI 1.58–2.95) and lower oxygen saturation (OR 1.64, 95%CI 1.24–2.19) were also associated with impaired cognition.

Diastolic blood pressure, mean arterial pressure, heart rate, pulse pressure, capillary refill and temperature were not associated with increased risk of cognitive impairment. Results were similar after exclusion of patients with missing 6-CIT score and those with minor trauma. When correcting for age, higher heart rate was associated with increased risk of cognitive impairment (lowest quartile vs highest quartile OR 1.48 (95%CI 1.10–1.98), p = 0.028). Table 2 shows that mean systolic blood pressure, mean arterial pressure, respiratory rate and oxygen saturation, differ between strata of cognitive function. Patients with more severe cognitive impairment had lower systolic blood pressure, lower respiratory rate and lower oxygen saturation.

**Table 2:** Vital signs over strata of cognitive function in older patients in the ED.

	Normal cognition n = 1561	Mild cognitive impairment n = 328	Cognitive impairment n = 125	Severe cognitive impairment n = 238	p for trend	Missing 6CIT n = 235	Pre-existing dementia n = 142
Systolic BP, mmHg	150 (28)	149 (28)	145 (28)	145 (28)	**0.017**	150 (30)	142 (28)
Diastolic BP, mmHg	80 (17)	79 (17)	78 (17)	79 (17)	0.121	80 (18)	75 (18)
MAP, mmHg	103 (17)	102 (18)	100 (18)	101 (18)	**0.032**	103 (19)	97 (18)
Heart rate/min	84 (22)	82 (21)	85 (23)	86 (21)	0.249	85 (21)	81 (22)
Respiratory rate/min	19 (6)	19 (5)	20 (7)	21 (6)	**<0.001**	20 (6)	19 (5)
Oxygen saturation, median (IQR)	97 (95–98)	97 (95–98)	96 (94–98)	96 (94–98)	**<0.001**	96 (94–98)	97 (95–98)
Temperature, celsius	36.9 (0.8)	37.0 (0.9)	37.0 (0.9)	36.8 (1.0)	0.901	36.9 (0.9)	36.9 (1.0)
Capillary refill, sec, median (IQR)	2 (2–3)	2 (2–3)	2 (2–3)	2 (2–3)	0.640	2 (2–3)	2 (2–3)

Data are presented as mean, SD unless noted otherwise.

Abbreviations: n = number, IQR = interquartile range, mmHg = millimetres of mercury, sec = seconds, min = minute, p = p value, BP = blood pressure, MAP = mean arterial pressure, ED = emergency department

Numbers between brackets indicate number of missing values: heart rate (n = 405), respiratory rate (n = 861), capillary refill (n = 1705), systolic BP (n = 375), diastolic BP (n = 379), saturation (n = 438), temperature (n = 716), MAP (n = 379).

Normal cognition = 6-CIT 0–7 points, mild cognitive impairment = 6-CIT 8–10 points, cognitive impairment = 6-CIT 11–13 points, severe cognitive impairment = 6-CIT ≥14 points, pre-existing dementia = dementia reported by patient or caregivers. Severe cognitive impairment largely represents patients with dementia.

P for trend was calculated over the following categories: normal cognition, mild cognitive impairment, cognitive impairment and severe cognitive impairment using linear regression.

### Association of laboratory test results with impaired cognition

**Fig 3:**
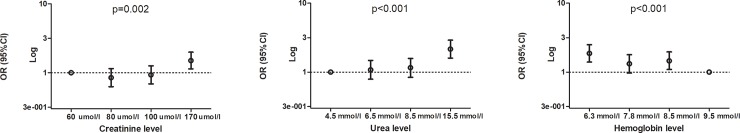
Quartiles of laboratory test results and their association with cognitive impairment. Abbreviations: umol/l = micromole per liter, mmol/l = millimole per liter, OR = odds ratio, 95%CI = 95% confidence interval, p = p-value. Y-axes is a logarithmic scale. The x-axes shows quartiles of the represented laboratory test result with the mean value within that category as label. The circle on the dotted line represents the reference category. Odds ratios and 95% CI’s can be found in S2 Table. Dot with bars represents the odds ratio with 95% confidence interval.

As can be seen in Fig 3, increased creatinine levels were associated with a higher chance of impaired cognition (OR 1.51, 95%CI 1.38–1.99), as were increased levels of urea (OR 2.16, 95%CI 1.61–2.91) and lower levels of haemoglobin (OR 1.87, 95%CI 1.40–2.49, S2 Table), this association was also seen when we corrected for age.

## Discussion

In older patients without pre-existing dementia, who present to the ED, cognitive impairment was associated with abnormalities associated with decreased brain perfusion and oxygenation, such as low systolic blood pressure, high respiratory rate and low oxygen saturation. There is also an association between laboratory test result that associate with decreased brain perfusion and oxygen delivery such as high urea, high creatinine and low haemoglobin and impaired cognition in this patient group.

Although the association between vital signs and impaired cognition has been studied in the long-term setting[16], our study suggests that in the ED setting this association also exists.

In chronic settings blood pressure variability, blood pressure and cardiac output associate with cognitive impairment in various patient populations[24–26]. Several studies found an association between hypoxia and cognitive impairment in the long-term setting[27, 28] Respiratory rate has one of the strongest associations with cognitive impairment, and is also strongly associated with sepsis, which is a syndrome characterized by decreased tissue oxygenation and perfusion[29]. There have been several studies which measured regional cerebral oxygenation in critically ill patients using near-infrared spectroscopy. A systematic review by Bendahan et al[17] showed that there may be a slight signal of association between low regional cerebral oxygenation and delirium[17], which is in line with our findings. Although the associations between strata of vital signs and cognitive impairment in our study are striking and a trend is seen (Fig 2), the mean differences in vital signs between the strata of cognitive impairment are quite small (Table 2). This means that creating cut-off points for high risk of cognitive impairment are difficult to choose and use in clinical practice at this point. For example, the systolic blood pressure of 145mmHg in the groups of patients with the most cognitive impairment would not trigger interventions or be considered abnormal in clinical practice. However, this is a hypothesis generating study creating a basis for further investigations.

In addition to vital signs reflecting acute respiratory and organ dysfunction, laboratory tests which are associated with tissue hypoperfusion and oxygen delivery, like creatinine, urea and haemoglobin are also associated with cognitive impairment in long-term settings. Siew et al. found that in ICU patients elevated levels of creatinine were associated with delirium and coma[30]. Also in patients with chronic end stage renal disease an association with cognitive impairment was found[31]. Overall, the association we find between vital signs and cognitive impairment in the acute setting seems similar to those found in chronic conditions.

Impaired cognition is a frequent finding in the ED setting, with an average prevalence of ~30% in the literature[3–7]. Although this may partially be caused by delirium and pre-existing dementia, a part of the prevalence of impaired cognition in the ED is unexplained. In this study we find a similar percentage of 28% cognitive impairment. The patients in this study are relatively independent with low Katz-ADL scores and few hours of home care, which might imply that these cognitive disorders are not so severe that they influence daily life or that these disorders might be transient. We hypothesize that in a proportion of patients with impaired cognition this may be related to transient compromised perfusion or oxygenation of the brain. We propose that not all patients with acute confusion and an underlying illness or abnormal vital signs have delirium. There may be another entity causing cognitive impairment, ‘brain hypoperfusion’. However, brain hypoperfusion could also be a cause of delirium in itself and it may be hypothesized that brain hypoperfusion results in mild cognitive impairment or subsyndromal delirium. This could be explained by several pathophysiological mechanisms: first, respiratory rate affects chemoregulation of the brain by changing arterial pCO2 and pH[19]. Second, cardiac output, arterial oxygen saturation and haemoglobin concentration determine oxygen delivery to the brain, potentially affecting cognitive function[32]. Finally, brain perfusion of older patients largely depends on adequate systolic and mean arterial pressures, due to adaptive cerebral vascular changes in old age leading to a shift of the lower limit of autoregulation towards high pressure, with an impaired tolerance to pressure decrease, explaining the association with cognitive function in the acute setting. Impaired brain perfusion and oxygen delivery may even result in local lactate accumulation in the brain, with a possible influence on cognitive function[18].

It should be stressed however that the observational character of the present study should leave room for other possible explanations of this association. First, it is possible that patients with pre-existing cognitive impairment present more ill to the ED because they alarm caregivers in later stages of disease.

Second, patients who are in distress, for example who suffer from dyspnea, which might be reflected by a high respiratory rate and low oxygen saturation, can focus less on the cognitive test and thereby have a worse score. Third, in the pathophysiological pathway of delirium there seems to be a role for inflammatory cytokines, cholinergic function and the so-called ‘aberrant stress response’[33], which might also mediate this association as severe illness such as sepsis, reflected by abnormal vital signs, can start this response of the body[32]. Finally, because both vital signs and delirium are associated with disease severity and mortality, they may reflect two sides of the same coin, rather than a causal relation.

Further studies are therefore necessary in which both brain perfusion/oxygenation and cognition are measured in the acute setting. In these studies a clear distinction between the different pathophysiological mechanisms, such as pre-existing cognitive impairment (i.e. dementia), intercurrent delirium, neurological disorders and brain hypoperfusion or combinations of these, should be made. A next step would then be to investigate the reversibility of impaired cognition by optimal resuscitation. Finally, it should be assessed whether clinically relevant endpoints such as functional decline and mortality improve if cognitive function is optimized in the acute setting.

This study has several limitations. First, cognition was tested within one hour after arrival to the ED. This could have influenced the cognition score. A patient who is anxious or in pain may perform worse resulting in an overestimation of the prevalence of impaired cognition. However, impaired cognition in older patients should always be a trigger for physicians to think further. Second, we did not perform any follow-up measurements of cognitive function and have no information about resuscitative efforts by the Emergency Medical Services or during the ED stay and the influence of this on vital signs. Third, we did not assess presence of delirium using gold standard assessment. Fourth, patients with need for immediate care and an unstable medical condition were excluded which could have led to selection bias. Finally, we do not have any measurements of cerebral blood flow. This would be a next step in studying this topic. Strengths of this study are the broad and unselected inclusion in several hospitals and the large sample size. This makes the conclusions more generalizable. Also the low number of missing data makes it possible to draw stronger conclusions. Finally, this is the first large multicentre study to investigate the relationship between vital sign abnormalities and cognitive impairment in the acute setting.

## Conclusions

In conclusion, we found an association between abnormal vital signs and cognitive impairment in older ED patients. Although this may partially reflect the association of disease severity with delirium, impaired cognition may also be caused by acute disturbance of brain perfusion and oxygenation. This is a first step towards further in-depth studies to investigate whether normalization of these vital signs will also improve brain perfusion and cognition.

Furthermore it emphasizes the importance for physicians to pay attention to cognition in the care for older ED patients.

## Supporting information

S1 Table Quartiles of vital signs and association with cognitive impairment, excluding patients with pre-existing dementia. Abbreviations: n = number, mmHg = millimetres of mercury, min = minute, % = percentage, ⁰ = degrees Celsius, sec = seconds, ref = reference category, OR = odds ratio, 95%CI = 95% confidence interval, p = p valueNumbers between brackets indicate missing values: respiratory rate (n = 812), heart rate (n = 383), systolic blood pressure (n = 354), diastolic blood pressure (n = 358), temperature (n = 672), oxygen saturation (n = 414), capillary refill (n = 1618), pulse pressure (n = 358), MAP (n = 358)Multivariable analysis is adjusted for age.
(DOCX)

S2 Table Quartiles of laboratory test results and association with cognitive impairment, excluding patients with pre-existing dementia. Abbreviations: n = number, umol/l = micromole per liter, mmol/l = millimole per liter, ref = reference category, OR = odds ratio, 95%CI = 95% confidence interval, p = p valueNumbers between brackets indicate missing values: creatinine (n = 500), urea (n = 696), haemoglobin (n = 472)Multivariable analysis is adjusted for age.
(DOCX)

S1 File APOP-database used in this study. In the S1 File the data used for this study can be found. For more information regarding the variables please contact the corresponding author.
(SAV)

## References

[1] Litovitz GL, Hedberg M, Wise TN, White JD, Mann LS. Recognition of psychological and cognitive impairments in the emergency department. The American journal of emergency medicine. 1985;3(5):400–2. Epub 1985/09/01. 10.1016/0735-6757(85)90197-4 . 4041189

[2] Schofield I, Stott DJ, Tolson D, McFadyen A, Monaghan J, Nelson D. Screening for cognitive impairment in older people attending accident and emergency using the 4-item Abbreviated Mental Test. European journal of emergency medicine: official journal of the European Society for Emergency Medicine. 2010;17(6):340–2. 10.1097/MEJ.0b013e32833777ab . 20164778

[3] Lucke JA, de Gelder J, Heringhaus C, van der Mast RC, Fogteloo AJ, Anten S, et al Impaired cognition is associated with adverse outcome in older patients in the Emergency Department; the Acutely Presenting Older Patients (APOP) study. Age and ageing. 2017:1–6. Epub 2017/11/28. 10.1093/ageing/afx174 . 29177470

[4] Carpenter CR, DesPain B, Keeling TN, Shah M, Rothenberger M. The Six-Item Screener and AD8 for the detection of cognitive impairment in geriatric emergency department patients. Annals of emergency medicine. 2011;57(6):653–61. 10.1016/j.annemergmed.2010.06.560 20855129 PMC3213856

[5] Gray LC, Peel NM, Costa AP, Burkett E, Dey AB, Jonsson PV, et al Profiles of older patients in the emergency department: findings from the interRAI Multinational Emergency Department Study. Annals of emergency medicine. 2013;62(5):467–74. 10.1016/j.annemergmed.2013.05.008 . 23809229

[6] Provencher V, Sirois MJ, Ouellet MC, Camden S, Neveu X, Allain-Boule N, et al Decline in activities of daily living after a visit to a canadian emergency department for minor injuries in independent older adults: are frail older adults with cognitive impairment at greater risk? Journal of the American Geriatrics Society. 2015;63(5):860–8. 10.1111/jgs.13389 . 25989564

[7] Schnitker LM, Beattie ER, Martin-Khan M, Burkett E, Gray LC. Characteristics of older people with cognitive impairment attending emergency departments: A descriptive study. Australasian emergency nursing journal: AENJ. 2016;19(2):118–26. Epub 2016/05/14. 10.1016/j.aenj.2016.04.002 . 27173359

[8] Elie M, Rousseau F, Cole M, Primeau F, McCusker J, Bellavance F. Prevalence and detection of delirium in elderly emergency department patients. CMAJ: Canadian Medical Association journal = journal de l'Association medicale canadienne. 2000;163(8):977–81. 11068569 PMC80546

[9] Hustey FM, Meldon SW. The prevalence and documentation of impaired mental status in elderly emergency department patients. Annals of emergency medicine. 2002;39(3):248–53. . 11867976 10.1067/mem.2002.122057

[10] Hare M, Wynaden D, McGowan S, Speed G. Assessing cognition in elderly patients presenting to the emergency department. International emergency nursing. 2008;16(2):73–9. 10.1016/j.ienj.2008.01.005 . 18519057

[11] Han JH, Zimmerman EE, Cutler N, Schnelle J, Morandi A, Dittus RS, et al Delirium in older emergency department patients: recognition, risk factors, and psychomotor subtypes. Academic emergency medicine: official journal of the Society for Academic Emergency Medicine. 2009;16(3):193–200. 10.1111/j.1553-2712.2008.00339.x . 19154565 PMC5015887

[12] Kennedy M, Enander RA, Tadiri SP, Wolfe RE, Shapiro NI, Marcantonio ER. Delirium risk prediction, healthcare use and mortality of elderly adults in the emergency department. Journal of the American Geriatrics Society. 2014;62(3):462–9. 10.1111/jgs.12692 24512171 PMC3959285

[13] Carpenter CR, Bassett ER, Fischer GM, Shirshekan J, Galvin JE, Morris JC. Four sensitive screening tools to detect cognitive dysfunction in geriatric emergency department patients: brief Alzheimer's Screen, Short Blessed Test, Ottawa 3DY, and the caregiver-completed AD8. Academic emergency medicine: official journal of the Society for Academic Emergency Medicine. 2011;18(4):374–84. 10.1111/j.1553-2712.2011.01040.x 21496140 PMC3080244

[14] Han JH, Bryce SN, Ely EW, Kripalani S, Morandi A, Shintani A, et al The effect of cognitive impairment on the accuracy of the presenting complaint and discharge instruction comprehension in older emergency department patients. Annals of emergency medicine. 2011;57(6):662–71 e2. 10.1016/j.annemergmed.2010.12.002 21272958 PMC3603343

[15] O'Sullivan D, Brady N, Manning E, O'Shea E, O'Grady S, N OR, et al Validation of the 6-Item Cognitive Impairment Test and the 4AT test for combined delirium and dementia screening in older Emergency Department attendees. Age and ageing. 2017:1–7. 10.1093/ageing/afx149 . 28985260 PMC5860384

[16] van Buchem MA, Biessels GJ, Brunner la Rocca HP, de Craen AJ, van der Flier WM, Ikram MA, et al The heart-brain connection: a multidisciplinary approach targeting a missing link in the pathophysiology of vascular cognitive impairment. J Alzheimers Dis. 2014;42 Suppl 4:S443–51. 10.3233/JAD-141542 . 25213769

[17] Bendahan N, Neal O, Ross-White A, Muscedere J, Boyd JG. Relationship Between Near-Infrared Spectroscopy-Derived Cerebral Oxygenation and Delirium in Critically Ill Patients: A Systematic Review. J Intensive Care Med. 2018:885066618807399. 10.1177/0885066618807399 . 30376764

[18] Bakker FC, Klijn CJ, Jennekens-Schinkel A, van der Tweel I, van der Grond J, van Huffelen AC, et al Cognitive impairment is related to cerebral lactate in patients with carotid artery occlusion and ipsilateral transient ischemic attacks. Stroke; a journal of cerebral circulation. 2003;34(6):1419–24. Epub 2003/04/26. 10.1161/01.STR.0000069725.09499.14 . 12714708

[19] Immink RV, Pott FC, Secher NH, van Lieshout JJ. Hyperventilation, cerebral perfusion, and syncope. J Appl Physiol (1985). 2014;116(7):844–51. Epub 2013/11/23. 10.1152/japplphysiol.00637.2013 . 24265279

[20] De Gelder J, Lucke J, De Groot B, Fogteloo AJ, Anten S, Mesri KS, E. W., et al Predicting adverse health outcomes in older emergency department patients: the APOP study. Netherlands Journal of Medicine. 2016;74(8):342–52. 27762216

[21] Mackway-Jones K. Manchester Triage Group. Emergency Triage. 1997.

[22] Tuijl JP, Scholte EM, de Craen AJ, van der Mast RC. Screening for cognitive impairment in older general hospital patients: comparison of the Six-Item Cognitive Impairment Test with the Mini-Mental State Examination. International journal of geriatric psychiatry. 2012;27(7):755–62. 10.1002/gps.2776 . 21919059

[23] Bianchi W, Dugas AF, Hsieh YH, Saheed M, Hill P, Lindauer C, et al Revitalizing a vital sign: improving detection of tachypnea at primary triage. Annals of emergency medicine. 2013;61(1):37–43. 10.1016/j.annemergmed.2012.05.030 . 22738682

[24] Sabayan B, Oleksik AM, Maier AB, van Buchem MA, Poortvliet RK, de Ruijter W, et al High blood pressure and resilience to physical and cognitive decline in the oldest old: the Leiden 85-plus Study. Journal of the American Geriatrics Society. 2012;60(11):2014–9. Epub 2012/11/07. 10.1111/j.1532-5415.2012.04203.x . 23126669

[25] Bornstein RA, Starling RC, Myerowitz PD, Haas GJ. Neuropsychological function in patients with end-stage heart failure before and after cardiac transplantation. Acta Neurol Scand. 1995;91(4):260–5. Epub 1995/04/01. . 7625151 10.1111/j.1600-0404.1995.tb07001.x

[26] Sabayan B, Wijsman LW, Foster-Dingley JC, Stott DJ, Ford I, Buckley BM, et al Association of visit-to-visit variability in blood pressure with cognitive function in old age: prospective cohort study. Bmj. 2013;347:f4600 Epub 2013/08/01. 10.1136/bmj.f4600 . 23900315

[27] Mikkelsen ME, Christie JD, Lanken PN, Biester RC, Thompson BT, Bellamy SL, et al The adult respiratory distress syndrome cognitive outcomes study: long-term neuropsychological function in survivors of acute lung injury. Am J Respir Crit Care Med. 2012;185(12):1307–15. Epub 2012/04/12. 10.1164/rccm.201111-2025OC 22492988 PMC3381234

[28] Kakkera K, Padala KP, Kodali M, Padala PR. Association of chronic obstructive pulmonary disease with mild cognitive impairment and dementia. Curr Opin Pulm Med. 2017 Epub 2017/12/13. 10.1097/MCP.0000000000000458 . 29232279

[29] Singer M, Deutschman CS, Seymour CW, Shankar-Hari M, Annane D, Bauer M, et al The Third International Consensus Definitions for Sepsis and Septic Shock (Sepsis-3). Jama. 2016;315(8):801–10. 10.1001/jama.2016.0287 26903338 PMC4968574

[30] Siew ED, Fissell WH, Tripp CM, Blume JD, Wilson MD, Clark AJ, et al Acute Kidney Injury as a Risk Factor for Delirium and Coma during Critical Illness. Am J Respir Crit Care Med. 2017;195(12):1597–607. Epub 2016/11/18. 10.1164/rccm.201603-0476OC 27854517 PMC5476907

[31] Kallenberg MH, Kleinveld HA, Dekker FW, van Munster BC, Rabelink TJ, van Buren M, et al Functional and Cognitive Impairment, Frailty, and Adverse Health Outcomes in Older Patients Reaching ESRD-A Systematic Review. Clin J Am Soc Nephrol. 2016;11(9):1624–39. Epub 2016/06/28. 10.2215/CJN.13611215 27342598 PMC5012494

[32] Sonneville R, Verdonk F, Rauturier C, Klein IF, Wolff M, Annane D, et al Understanding brain dysfunction in sepsis. Ann Intensive Care. 2013;3(1):15 Epub 2013/05/31. 10.1186/2110-5820-3-15 23718252 PMC3673822

[33] Maclullich AM, Anand A, Davis DH, Jackson T, Barugh AJ, Hall RJ, et al New horizons in the pathogenesis, assessment and management of delirium. Age and ageing. 2013;42(6):667–74. 10.1093/ageing/aft148 24067500 PMC3809721

