# Supplementary material for: Vital signs and impaired cognition in older emergency department patients: The APOP study
Source: PLoS One. 2019 Jun 20;14(6):e0218596. doi: 10.1371/journal.pone.0218596 (PMC6586336; doi:10.1371/journal.pone.0218596)
Supplement: S1 Table — Abbreviations: n = number, mmHg = millimetres of mercury, min = minute, % = percentage, ⁰ = degrees Celsius, sec = seconds, ref = reference category, OR = odds ratio, 95%CI = 95% confidence interval, p = p valueNumbers between brackets indicate missing values: respiratory rate (n = 812), heart rate (n = 383), systolic blood pressure (n = 354), diastolic blood pressure (n = 358), temperature (n = 672), oxygen saturation (n = 414), capillary refill (n = 1618), pulse pressure (n = 358), MAP (n = 358)Multivariable analysis is adjusted for age. (DOCX) [file pone.0218596.s001.docx]

**Supplemental table S1:** Quartiles of vital signs and association with cognitive impairmen**t,** excluding patients with dementia

| Range | Patients per quartile  total n=2487 | Univariable  (OR, 95% CI) | p for trend | Multivariable (OR, 95% CI) | p for trend |
| --- | --- | --- | --- | --- | --- |
| **Vital signs** |  |  |  |  |  |
| Systolic blood pressure |  |  | **0.038** |  | **0.015** |
| 61-128 mmHg | 512 | 1.30 (0.98-1.73) |  | 1.40 (1.05-1.87) |  |
| 129-147 mmHg | 553 | 1.29 (0.98-1.70) |  | 1.39 (1.05-1.85) |  |
| 148-165 mmHg | 521 | 1.11 (0.84-1.48) |  | 1.24 (0.92-1.66) |  |
| 166-257 mmHg | 547 | ref |  | ref |  |
| Diastolic blood pressure |  |  | 0.265 |  | 0.406 |
| 15-67 mmHg | 496 | 1.18 (0.89-1.57) |  | 1.12 (0.84-1.50) |  |
| 68-78 mmHg | 536 | 1.09 (0.82-1.44) |  | 1.07 (0.80-1.42) |  |
| 79-89 mmHg | 552 | 1.08 (0.82-1.42) |  | 1.02 (0.77-1.36) |  |
| 90-187 mmHg | 545 | ref |  | ref |  |
| Mean Arterial Pressure |  |  | 0.135 |  | 0.109 |
| 45-90 mmHg | 521 | 1.18 (0.89-1.56) |  | 1.20 (0.91-1.60) |  |
| 90-101 mmHg | 534 | 1.17 (0.88-1.54) |  | 1.23 (0.92-1.63) |  |
| 102-113 mmHg | 531 | 0.98 (0.74-1.30) |  | 1.03 (0.77-1.37) |  |
| 114-205 mmHg | 543 | ref |  | ref |  |
| Pulse Pressure |  |  | 0.834 |  | 0.327 |
| 10-52 mmHg | 535 | 1.07 (0.81-1.41) |  | 1.17 (0.88-1.56) |  |
| 53-67 mmHg | 540 | 0.96 (0.72-1.27) |  | 1.09 (0.82-1.45) |  |
| 68-83 mmHg | 520 | 1.07 (0.81-1.41) |  | 1.13 (0.85-1.50) |  |
| 84-177 mmHg | 534 | ref |  | ref |  |
| Heart rate |  |  | 0.056 |  | **0.028** |
| 28-69/min | 502 | ref |  | ref |  |
| 70-79/min | 494 | 1.22 (0.91-1.64) |  | 1.22 (0.90-1.64) |  |
| 80-94/min | 588 | 1.09 (0.82-1.45) |  | 1.07 (0.80-1.43) |  |
| 95-205/min | 520 | 1.40 (1.05-1.86) |  | 1.48 (1.10-1.98) |  |
| Respiratory rate |  |  | **<0.001** |  | **<0.001** |
| 6-15/min | 423 | ref |  | ref |  |
| 16-18/min | 487 | 1.06 (0.76-1.48) |  | 0.99 (0.71-1.40) |  |
| 19-21/min | 296 | 1.47 (1.02-2.11) |  | 1.42 (0.99-2.05) |  |
| 22-64/min | 469 | 2.16 (1.58-2.95) |  | 1.96 (1.43-2.70) |  |
| Oxygen saturation |  |  | **<0.001** |  | **<0.001** |
| 73-95% | 604 | 1.64 (1.24-2.19) |  | 1.63 (1.22-2.18) |  |
| 96% | 330 | 1.08 (0.76-1.51) |  | 1.06 (0.75-1.50) |  |
| 97-98% | 714 | 0.87 (0.65-1.17) |  | 0.90 (0.67-1.22) |  |
| 99-100% | 425 | ref |  |  |  |
| Temperature |  |  | 0.967 |  | 0.951 |
| 32.7-36.3⁰ | 410 | ref |  | ref |  |
| 36.4-36.8⁰ | 506 | 0.80 (0.59-1.08) |  | 0.86 (0.64-1.17) |  |
| 36.9-37.3⁰ | 468 | 0.82 (0.60-1.11) |  | 0.82 (0.60-1.12) |  |
| 37.4-40.3⁰ | 431 | 0.99 (0.73-1.34) |  | 1.02 (0.75-1.39) |  |
| Capillary refill |  |  | 0.115 |  | 0.096 |
| 1-1.5 sec | 147 | ref |  | ref |  |
| 2.0 sec | 427 | 0.63 (0.41-0.95) |  | 0.57 (0.37-0.87) |  |
| 2.5 sec | 41 | 0.45 (0.19-1.10) |  | 0.37 (0.15-0.91) |  |
| 3.0-6.0 sec | 254 | 0.62 (0.39-0.98) |  | 0.59 (0.37-0.93) |  |

- Abbreviations: n=number, mmHg=millimetres of mercury, min=minute, %=percentage, ⁰=degrees Celsius, sec=seconds, ref= reference category, OR=odds ratio, 95%CI=95% confidence interval, p=p value
- Numbers between brackets indicate missing values: respiratory rate (n=812), heart rate (n=383), systolic blood pressure (n=354), diastolic blood pressure (n=358), temperature (n=672), oxygen saturation (n=414), capillary refill (n=1618), pulse pressure (n=358), MAP (n=358)
- Multivariable analysis is adjusted for age
