# Supplementary material for: Vital signs and impaired cognition in older emergency department patients: The APOP study
Source: PLoS One. 2019 Jun 20;14(6):e0218596. doi: 10.1371/journal.pone.0218596 (PMC6586336; doi:10.1371/journal.pone.0218596)
Supplement: S2 Table — Abbreviations: n = number, umol/l = micromole per liter, mmol/l = millimole per liter, ref = reference category, OR = odds ratio, 95%CI = 95% confidence interval, p = p valueNumbers between brackets indicate missing values: creatinine (n = 500), urea (n = 696), haemoglobin (n = 472)Multivariable analysis is adjusted for age. (DOCX) [file pone.0218596.s002.docx]

**Supplemental table S2:** Quartiles of laboratory test results and association with cognitive impairment

| Range | Patients per quartile  total n=2487 | Univariable  (OR, 95% CI) | p for trend | Multivariable (OR, 95% CI) | p for trend |
| --- | --- | --- | --- | --- | --- |
| **Laboratory test results** |  |  |  |  |  |
| Creatinine |  |  | **0.002** |  | **0.016** |
| 5-68 umol/l | 490 | ref |  | ref |  |
| 69-86 umol/l | 508 | 0.85 (0.63-1.15) |  | 0.82 (0.60-1.10) |  |
| 87-110 umol/l | 490 | 0.93 (0.69-1.26) |  | 0.89 (0.66-1.20) |  |
| 111-833 umol/l | 499 | 1.51 (1.38-1.99) |  | 1.38 (1.03-1.83) |  |
| Urea |  |  | **<0.001** |  | **<0.001** |
| 2.0-5.7 mmol/l | 460 | ref |  | ref |  |
| 5.8-7.2 mmol/l | 452 | 1.08 (0.79-1.48) |  | 0.99 (0.72-1.36) |  |
| 7.3-9.9 mmol/l | 441 | 1.16 (0.84-1.58) |  | 1.02 (0.74-1.41) |  |
| 10.0-100 mmol/l | 438 | 2.16 (1.61-2.91) |  | 1.79 (1.32-2.43) |  |
| Haemoglobin |  |  | **<0.001** |  | **0.007** |
| 2.2-7.2 mmol/l | 504 | 1.87 (1.40-2.49) |  | 1.64 (1.22-2.20) |  |
| 7.3-8.0 mmol/l | 471 | 1.32 (0.97-1.79) |  | 1.13 (0.83-1.55) |  |
| 8.1-8.8 mmol/l | 528 | 1.46 (1.09-1.96) |  | 1.38 (1.02-1.86) |  |
| 8.9-11.0 mmol/l | 512 | ref |  | ref |  |

- Abbreviations: n=number, umol/l=micromole per liter, mmol/l=millimole per liter, ref= reference category, OR=odds ratio, 95%CI=95% confidence interval, p=p value
- Numbers between brackets indicate missing values: creatinine (n=500), urea (n=696), haemoglobin (n=472)
- Multivariable is corrected for age
